# Adsorption of tetracycline on Fe (hydr)oxides: effects of pH and metal cation (Cu^2+^, Zn^2+^ and Al^3+^) addition in various molar ratios

**DOI:** 10.1098/rsos.171941

**Published:** 2018-03-28

**Authors:** Liang-Ching Hsu, Yu-Ting Liu, Chien-Hui Syu, Mei-Hsia Huang, Yu-Min Tzou, Heng Yi Teah

**Affiliations:** 1 Scientific Research Division, National Synchrotron Radiation Research Center, 101 Hsin-Ann Road, Hsinchu 300, Taiwan, Republic of China; 2 Department of Soil and Environmental Sciences, National Chung-Hsing University, 145 Xingda Road, Taichung 402, Taiwan, Republic of China; 3 Division of Agricultural Chemistry, Taiwan Agricultural Research Institute, 189 Zhongzheng Road, Wufeng District, Taichung 41362, Taiwan, Republic of China; 4 Division of Environmental Studies, Graduate School of Frontier Sciences, University of Tokyo, 332 Building of Environmental Studies, 5-1-5 Kashiwanoha, Kashiwa, Chiba 277-8563, Japan

**Keywords:** tetracycline, Fe (hydr)oxide, metal cations, molar ratio, adsorption

## Abstract

Iron (Fe) (hydr)oxides control the mobility and bioavailability of tetracycline (TC) in waters and soils. Adsorption of TC on Fe (hydr)oxides is greatly affected by polyvalent metals; however, impacts of molar metal/TC ratios on TC adsorptive behaviours on Fe (hydr)oxides remain unclear. Results showed that maximum TC adsorption on ferrihydrite and goethite occurred at pH 5–6. Such TC adsorption was generally promoted by the addition of Cu^2+^, Zn^2+^ and Al^3+^. The greatest increase in TC adsorption was found in the system with molar Cu/TC ratio of 3 due to the formation of Fe hydr(oxide)–Cu–TC ternary complexes. Functional groups on TC that were responsible for the complexation with Cu^2+^shifted from phenolic diketone groups at Cu/TC molar ratio < 1 to amide groups at Cu/TC molar ratio ≥ 1. For the addition of Al^3+^, the complexation only took place with phenolic diketone groups, resulting in the enhanced TC adsorption at a molar Al/TC ratio of 1. However, TC adsorption decreased for Al/TC molar ratio > 1 as excess Al^3+^ led to the competitive adsorption with Al/TC complexes. For the Zn^2+^ addition, no significant correlation was found between TC adsorption capacity and molar Zn/TC ratios.

## 1. Introduction

Up to 13.5 million kilograms of antimicrobial chemicals were sold in the USA in 2011, and 80% of these chemicals were antibiotics, which were applied in livestock production as non-therapeutical administration [1]. Tetracycline (TC) is one of the most widely used antibiotics, which is responsible for around 40% of antimicrobial application in swine production in the USA [2,3]. Additionally, TC is commonly applied in human infection medicines, veterinary medicines and animal growth promoters. Because only small amounts of TC would be absorbed in the digestive tract, up to 50–80% TC is excreted unmetabolized via faeces and urine [4,5]. Since animal wastes or manures are directly discharged to drainage and applied as fertilizers in agriculture activities, TC might be widely distributed over waters and soils. Owing to the absence of efficient adsorbents, TC is relatively preserved and/or mobile in these environments, resulting in the dissemination of antibiotic-resistant pathogens that threaten biota and disrupt indigenous microbial populations [6–9].

TC is an amphoteric molecule with three pKa values (3.3, 7.7 and 9.7; electronic supplementary material, figure S1). Thus, TC exists as a cationic, zwitterionic and anionic species under acidic, curcim-neutral and alkaline conditions, respectively. Under acidic conditions, the occurrence of a reversible epimerization at the position of C-4, forming 4-epi-TC, results in the relatively high solubility of TC [10]. At the pH range of 6–7.7 that is typically found in ecosystems, the predominant zwitterionic forms of TC tend to aggregate in solution, particularly in the presence of divalent cations, such as Ca^2+^ and Mg^2+^ [11,12]. In addition, spectroscopic results indicated that divalent metals tended to bond with hydroxyl groups (pKa_2_) on TC at pH > 7, wherein hydroxyl groups were more negatively charged and the C-4 nitrogen (pKa_3_) started to deprotonate [13,14]. Accordingly, pH and metal types are two major factors controlling the metal/TC complexation, and subsequently affecting aqueous solubility, molecular conformation and gastrointestinal absorption of TC [13,15]. Chemical structures of TC give a clue not only to the reactivity in solutions but also to the interaction with colloids and soil components. For example, at acidic conditions, the positively charged TC might undergo a cation exchange reaction with negatively charged surfaces of clay particles. Nonetheless, electrostatic repulsions between negatively charged TC and clay particles under alkaline conditions usually lead to the reduction of TC adsorption unless multivalent metals are present.

Components of aqueous colloids including clays, organoclays and humic substances are able to sorb TC via the primary mechanism of outer sphere interaction at acidic conditions [16–19]. Given that adsorption on colloids is a promising approach to regulate the mobility and bioavailability of TC in waters, the quantities and types of metals that often exist in aqueous environments due to anthropogenic activities should be considered to accurately predict the adsorption behaviours of TC. The molecular conformation of TC might change while complexed with metals. For instance, metal cations, such as Cu^2+^ and Cd^2+^, could enhance TC adsorption on kaolinite [18], montmorillonite [12,20] and soils/sediments [17]. The enhancement of TC adsorption was attributed to the metal–TC complexation, wherein the metal cations formed surface bridges between TC and adsorbents [20,21]. On the other hand, some divalent electrolyte cations, such as Ca^2+^ and Mg^2+^, inhibited TC adsorption on kaolinite over the pH range of 3–9 [18] even if the complexation of these two cations with TC had also been reported by Lambs *et al*. [13]. Competitive adsorption of Ca^2+^ and Mg^2+^ with positively charged TC or metal–TC complexes in the acidic condition combined with the lack of ion bridging effect, i.e. forming surface ternary bridging species, at an alkaline condition may explain the decreasing TC adsorption on kaolinite. Figueroa *et al*. [22] also found that, at low pH, Ca^2+^ inhibited TC adsorption on montmorillonite surfaces due to the occurrence of competitive adsorption; however, they reported a metal bridging effect between Ca and TC, which enhanced TC adsorption at alkaline conditions.

Iron (hydr)oxides, such as goethite and ferrihydrite, are widely distributed in ecosystems. These minerals may contribute to TC adsorption upon their solubilization, resulting in the probable formation of Fe–TC complexes [11,23]. Figueroa and Mackay [24] reported that goethite, haematite and oxide-rich soils exhibited a high affinity to TC compounds due to the surface complexation process even in an unfavourable electrostatic condition. Such results agree with what Gu & Karthikeyan [25] reported: TC promoted the dissolution of Fe hydroxides because the tricarbonylamide and carbonyl groups formed inner-sphere complexes with Fe. Although Zhao *et al*. [18] indicated that 0.01 mM electrolyte cations, including Li^+^, Na^+^, K^+^, Ca^2+^ and Mg^2+^, exhibited little effect on TC adsorption on goethite over the pH range of 3–10, a relatively significant enhancement of TC adsorption on goethite was observed in the presence of Cu^2+^ due to the formation of TC–Cu^2+^–goethite surface complexes. Given that metal cations usually accompany TC to occur in environments, it is necessary to take metal cations into account in regulating TC adsorption on aqueous colloids, fluvial sediments and soils.

Compared to the well-developed adsorptive behaviours of TC compounds on clay minerals, the interactions of TC with oxide surfaces as affected by various metal cations in relation to the molar metal/TC ratios remain unclear. This study aimed to determine TC adsorption on ferrihydrite (5Fe_2_O_3_•9H_2_O) and goethite (α-FeOOH), representative of amorphous and crystalline Fe (hydr)oxides, in the presence of Cu^2+^, Zn^2+^ and Al^3+^. Effects of various metal/TC molar ratios at different pH were systemically investigated in this study.

## 2. Material and methods

### 2.1. Reagents

Chemicals and reagents used here were all of analytical grade. The hydrochloride salt of tetracycline (96% pure) was purchased from Sigma-Aldrich Co. Other chemicals, including Fe(NO_3_)_3_, ZnCl_2_, CuCl_2_, AlCl_3_, NaCl, HCl and NaOH, were obtained from Merck. All solutions were prepared using 18 MΩ deionized water. All reaction vessels were acid-washed using 1 M HCl and rinsed with deionized water prior to the experiments. All experiments were carried out in a 0.01 M NaCl background, and the pH values were adjusted using 0.01 M HCl or NaOH.

### 2.2. Preparation and characterizations of Fe (hydr)oxides

Goethite was synthesized by mixing 1 M Fe(NO_3_)_3_ to 5 M NaOH and incubating the suspension for 60 h at 70°C [26]. The 2-line ferrihydrite was prepared by slowly adding 200 ml of 1 M NaOH into 500 ml of 0.1 M Fe(NO_3_)_3_ to obtain a final pH of 7–8 with stirring [26].

A subsample of each suspension was washed with deionized water to remove excess salts and freeze-dried prior to analyses of powder X-ray diffraction (XRD), point of zero charge (PZC), particle size distribution and specific surface areas. Details of preparation and characterizations are shown in the electronic supplementary material.

### 2.3. pH-dependent adsorption of tetracycline on Fe (hydr)oxides

Suspensions of goethite or ferrihydrite were mixed with certain amounts of freshly prepared TC stock solution (0.225 mM) in a 50 ml brown glass vial to achieve an initial TC concentration of 0.0225 mM and solid concentrations of 0.1 and 0.05 g l^−1^ for goethite and ferrihydrite, respectively. The pH values of mixed suspensions were adjusted to 3–9 using 0.01 M NaOH or HCl. Vials with Teflon liners were continuously shaken at150 r.p.m. and 24.5 ± 0.5°C. After 48 h, suspensions were passed through a 0.2 µm pore-sized filter membrane (cellulose ester, Chrom Tech Inc.) using a glass syringe. The TC concentrations in filtrates were determined using UV–visible spectroscopy (Cary 50, Varian Inc.) at 357 nm, an isobestic point of TC over the tested pH. Adsorbed TC was calculated from the differences between initial and final concentrations in solutions and filtrates. Photolysis of TC was negligible over the course of incubation (see the electronic supplementary material).

### 2.4. Adsorption isotherm of tetracycline on Fe (hydr)oxides

Adsorption isotherms of TC on goethite or ferrihydrite with and without the addition of metal cations (Zn^2+^, Cu^2+^ and Al^3+^) were conducted using the batch mode with three replicates at pH 3–6. Suspensions containing 0.1 or 0.05 g l^−1^ of goethite or ferrihydrite were mixed with 0.0113–0.113 mM TC solutions. In the isotherms with metal cations, TC was added at a molar metal/TC ratio of 1 after Zn^2+^, Cu^2+^ or Al^3+^ reacted with adsorbents for 24 h. Other experimental details were the same as those for the system without metal cations addition. All suspensions were continuously shaken for 48 h at 24.5 ± 0.5°C, and then passed through a 0.2 µm pore-sized filter membrane. The TC or metal/TC complexes in filtrates were determined by UV–visible spectroscopy at 357, 365 or 378 nm, in accordance with the greatest absorbance for individual metal/TC complexes [27]. In the preliminary test, the red shift of TC was found from 357 nm to 365 and 378 nm while TC reacted with Cu and Al in a 1 : 1 molar ratio (electronic supplementary material, figure S2). For the Zn/TC samples, however, no red shift was found. Therefore, calibration curves for Cu/TC and Al/TC systems were developed at 365 and 378 nm.

### 2.5. Adsorption kinetics of tetracycline on Fe (hydr)oxides

Kinetics experiments were carried out with initial TC concentration of 0.0225 mM and solid concentrations of 0.1 and 0.05 g l^−1^ for goethite and ferrihydrite. Amounts of 250 ml of suspensions were prepared in 500 ml jacketed borosilicate glass beakers, the pH of which was controlled at 3, 5 or 8 during 48 h incubation. Effects of metal cation (Zn^2+^, Cu^2+^ and Al^3+^) on the adsorption kinetics were also investigated. The experiments were performed via three manners: (i) metal cations and TC at a molar ratio of 1 : 1 (each 0.0225 mM) reacted first for 24 h to form complexes and then mixed with suspensions of goethite or ferrihydrite; (ii) 0.0225 mM of metal cations were first reacted with goethite or ferrihydrite for 1 h, and then 0.0225 mM of TC was added into the suspensions; and (iii) metal cations reacted with TC at fixed TC concentration of 0.0225 mM and molar metal/TC ratios of 0.1, 0.2, 1, 3, 5, 8 and 10 for 24 h and then mixed with goethite or ferrihydrite suspensions. For all three systems, the total equilibration duration was 48 h and the final solid concentration for goethite and ferrihydrite was 0.1 and 0.05 g l^−1^ (the total volume of each system was 250 ml). At each sampling point, 10 ml of suspensions were collected and passed through a 0.2 µm pore-sized filter membrane. Amounts of TC or metal/TC complexes in filtrates were determined by UV–visible spectroscopy at 357, 365 and 378 nm for TC only and Zn/TC, Cu/TC, as well as Al/TC systems, respectively. Precipitates collected from the third kinetic treatment mentioned above were analysed using a Fourier transform infrared (FTIR) spectrometer (Thermo Nicolet NEXUS 470). Mixtures of 0.001 g well-grounded freeze-dried samples with 0.2 g infrared-grade KBr were compressed as pellets. Spectra were collected at an optical resolution of 4 cm^−1^. For each sample, 64 individual scans were acquired in the range of 4000–500 cm^−1^ and merged using the GRAMS AI program.

## 3. Results

### 3.1. Characterizations of Fe (hydr)oxides

The synthesized 2-line ferrihydrite and goethite were characterized using XRD analysis (electronic supplementary material, figure S3). The pH_pzc_ of ferrihydrite and goethite are 6.3 and 6.5. The mean particle sizes of ferrihydrite and goethite are 290.3 and 466.5 nm. The specific surface areas of ferrihydrite and goethite are 251.8 and 25 m^2^ g^−1^.

### 3.2. pH-dependent adsorption of tetracycline on Fe (hydr)oxides

**Figure 1.**
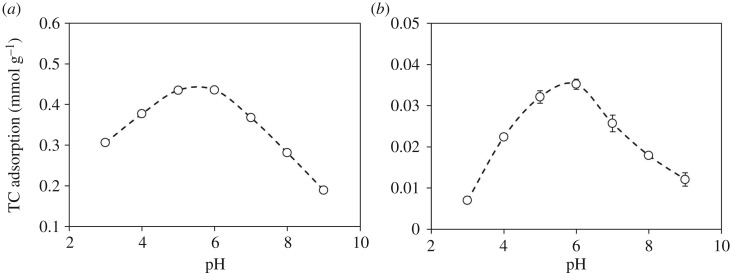
The pH-dependent adsorption of TC on (*a*) ferrihydrite and (*b*) goethite.

Adsorption of TC on ferrihydrite and goethite as a function of pH is shown in figure 1. The adsorption amounts of TC on both adsorbents increased from pH 3–5, reached the maximum at pH 5–6, and decreased for pH > 6. Such adsorption behaviour could be attributed to the electrostatic interactions between pH-dependent charges of TC molecules and surface charges of Fe (hydr)oxides. Like many veterinary antibiotics, TC exhibits multiple ionizable functional groups including dimethylammonium, tricarbonylamide and phenolic diketone groups (electronic supplementary material, figure S1). These functional groups undergo protonation–deprotonation reactions, forming cation species of H_2_TC^+^ at pH < 3.3, zwitterion species of H_2_TC^0^ between pH 3.3 and 7.7, or anion species of HTC^−^/TC^2−^ at pH > 7.7. At pH 5–6, the major species of TC was zwitterionic, and the net surface charges of the Fe (hydr)oxides were slightly positive (pH_zpc_ = 6.3–6.5, electronic supplementary material, table S1). Although TC molecules were electrically neutral at pH 5–6, the electrostatic attraction may occur between the negatively charged tricarbonylamide groups and the positively charged surfaces of Fe (hydr)oxides. Thus, the maximum adsorption capacity of TC on Fe hydr(oxides) was found at pH 5–6. When the pH was lower than 5–6, the electrostatic repulsion between the cationic moieties of TC and positive charges on the surfaces of Fe (hydr)oxides led to a decrease in TC adsorption. When the system pH was higher than the PZC of Fe (hydr)oxides (pH 6.3 and 6.5 for ferrihydrite and goethite), the emergence of negative charges on the surfaces of Fe (hydr)oxides repulsed the anionic TC, resulting in a decrease in TC adsorption. That is, both positive–positive and negative–negative repulsion took place in the TC/Fe (hydr)oxide systems. Thus, a favourable electrostatic attraction between TC and Fe (hydr)oxides was observed at the intermediate pH range, causing a bell-shaped adsorption curve as a function of pH with the maximum TC adsorption at the pH close to the pH_pzc_ of the adsorbents [18,25,28,29]. The other noteworthy point was that amounts of TC adsorbed onto ferrihydrite were approximately 10-fold greater than that on goethite, suggesting the contribution of higher surface areas and smaller particle size of ferrihydrite to TC adsorption.

### 3.3. Adsorption isotherm of tetracycline on Fe (hydr)oxides as affected by metal cations (Cu^2+^, Zn^2+^ and Al^3+^) at various pH

**Figure 2.**
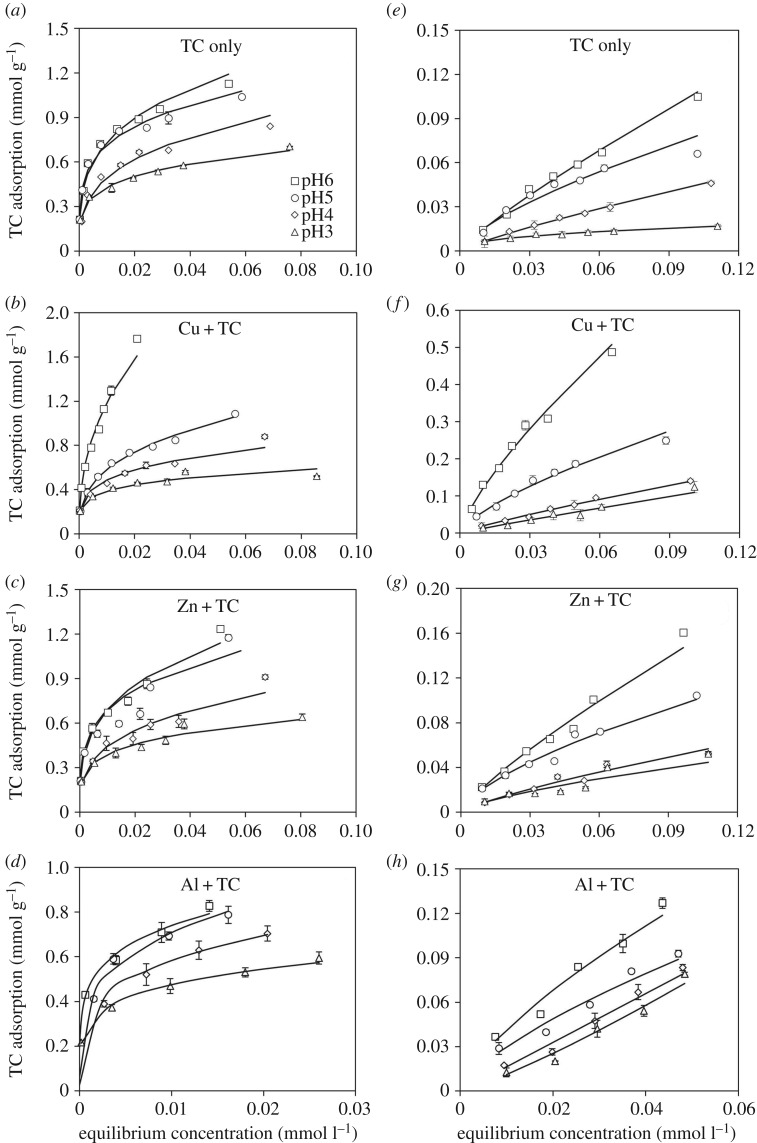
Adsorption isotherms of TC on (*a–d*) ferrihydrite and (*e–h*) goethite at pH 3–6 as affected by the addition of Cu, Zn and Al. TC was added at a molar metal/TC ratio of 1 after Zn^2+^, Cu^2+^ or Al^3+^ reacted with adsorbents for 24 h. The equilibration duration was 48 h. Solid lines represent the fitted Freundlich data.

The adsorption isotherms of TC on ferrihydrite and goethite as influenced by metal cation additions (Cu^2+^, Zn^2+^ and Al^3+^) at various pH are shown in figure 2. The isotherm data were all adequately described by the Freundlich isotherm model, and the related parameters are listed in electronic supplementary material, table S2. Wang *et al*. [20] as well as Gu & Karthikeyan [25] also indicated that the TC adsorption on Fe/Al hydroxides and montmorillonite could be well described by the Freundlich model. However, Figueroa & Mackay [24] suggested that the TC adsorption on goethite was adequately described by the Langmuir model. Such discrepancy might be caused by different preparation methods for the goethite. Given that our goethite was freshly prepared and stored as suspensions at 4°C, its degree of aggregation could be alleviated while compared with that used in Figueroa & Mackay [24], wherein the goethite was a commercial product and dried as powder. As a consequence, our goethite showed a greater surface area (25.0 m^2^ g^−1^) than that used in Figueroa & Mackay [24] (17.8 m^2^ g^−1^).

The Freundlich parameters of *K*_f_ and 1/*n* represent the distribution coefficient and adsorption intensity, respectively. The lower 1/*n* value (close to 1) indicates a more heterogeneous system and a favourable sorption process. Once the 1/*n* value is less than 1, adsorption showed a nonlinear curve, and the adsorbed increments tended to decrease with increasing TC concentrations in equilibration, suggesting that adsorption sites on Fe (hydr)oxides were not homogeneous [25,30]. In the absence of metal cations, the *K*_f_ for TC adsorbed on ferrihydrite (1.202–2.763 mg^1−1/*n*^ l^1/*n*^ g^−1^) were higher than that on goethite (0.141–0.759 mg^1−1/*n*^ l^1/*n*^ g^−1^). On both adsorbents, the K_f_ increased with increasing pH from 3 to 6, agreeing with the pH-dependent adsorption results shown in the electronic supplementary material, figure S1.

Compared with the pure TC system, the addition of Cu^2+^, Zn^2+^ and Al^3+^ resulted in a slight decrease in TC adsorption on ferrihydrite at pH 3 and 4 (figure 2*b–d*). At pH 6, however, TC adsorption on ferrihydrite was essentially boosted with the addition of Cu^2+^ (figure 2*b*). In terms of Al^3+^, it promoted the TC adsorption on ferrihydrite at pH 3–5 but not at pH 6 according to the *K*_f_ values (electronic supplementary material, table S2). Unlike the ferrihydrite system, the addition of metal cations generally enhanced the TC adsorption on goethite among all tested pH while compared with the pure TC system (figure 2*e–h*). Wherein, Cu^2+^ addition resulted in the most significant increase in TC adsorption, particularly at pH 5 and 6.

Collectively, Cu^2+^ showed the greatest ability to promote the TC adsorption on Fe (hydr)oxides at pH 5–6. Such enhancement in TC adsorption could be ascribed to the cationic bridging effect resulting from the ligand-to-metal charge transfer that formed Cu–TC complexes with high affinity to Fe (hydr)oxide surfaces [29,31]. The formation of Cu–TC complexes at a slightly acidic pH, e.g. pH 5, could be evidenced by a red-shift for the absorption peak of TC from 357 to 365 nm (electronic supplementary material, figure S2) [32]. However, the Cu–TC complexation was restricted under more acidic conditions. For instance, Zhao *et al*. [33] reported that no spectral red shift for TC/Cu^2+^ system was found at pH ≤ 4. The absence of Cu–TC complex might possibly account for the indiscernible impact of Cu^2+^ addition on TC adsorption on Fe (hydr)oxides at pH 3 and 4. Meanwhile, the free Cu^2+^ might compete with TC for the adsorption sites and thus impede the TC adsorption. At the same pH range, however, TC adsorption on Fe (hydr)oxdies could be promoted when Al^3+^ was added, indicating the complexation between TC and trivalent cations was relatively favourable at the acidic conditions. On the contrary, the enhancement in TC adsorption on ferrihydrite accompanying the Al^3+^ addition seemed to be negligible at pH 6. That is because a certain proportion of Al might hydrolyse as Al hydroxides and thus hinder the complexation between Al and TC even if the newly formed Al(OH)_3_ is promising to retain TC [28]. In terms of Zn^2+^ addition on the goethite system, we do not yet fully understand what factors controlled the enhancement of TC adsorption on goethite at circumneutral pH because no significant spectroscopic evidences suggested that Zn–TC complexes were formed in this study (electronic supplementary material, figure S2).

### 3.4. Adsorption kinetics of tetracycline on Fe (hydr)oxides

**Figure 3.**
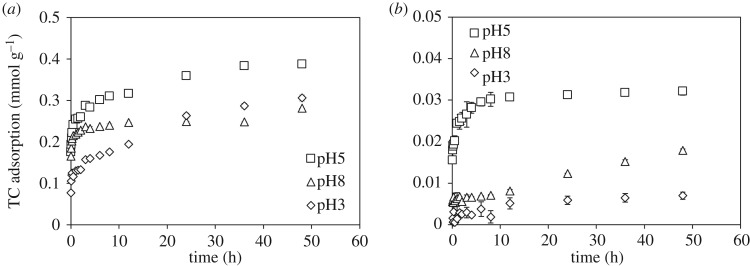
Adsorption kinetics of TC on (*a*) ferrihydrite and (*b*) goethite at pH 3, 5 and 8.

The adsorption kinetics of TC on ferrihydrite and goethite at pH 3, 5 and 8 (figure 3) was adequately fitted by the pseudo-second-order kinetic model, which has been applied widely to describe the chemisorption of pollutants from aqueous solutions on adsorbents [34]. The corresponding kinetic parameters are listed in the electronic supplementary material, table S3, wherein the *k* and *q*_e_ parameters represented the adsorption rate constants and the amounts of TC adsorbed on Fe (hydr)oxides at equilibration, respectively. For both adsorbents, *k* and *q*_e_ had maximum values at pH 5 (electronic supplementary material, table S3), indicating that the TC not only exhibited a stronger affinity on these two Fe (hydr)oxides but also adsorbed more rapidly at this specific pH value. The pH-dependent changes in *k*-values for the TC adsorption on goethite varied from 3.69 to 47.33 g mmol^−1^ h^−1^, which were significantly greater than 0.691–2.351 g mmol^−1^ h^−1^ obtained in the ferrihydrite system (electronic supplementary material, table S3). Although the amounts of TC adsorbed on goethite were lower than that on ferrihydrite as evidenced by the *q*_e_ values, the rapid saturation of the limited adsorption sites and the lack of efficient internal sites for continuous adoption may facilitate TC adsorption on goethite with well-crystalline structures. Zhao *et al*. [18] reported that TC formed surface complexes on surfaces of goethite, which also followed the pseudo-second-order kinetic model. For the TC adsorption on ferrihydrite, reactions took place relatively fast in the initial half hour, followed by slow increments of TC adsorption with prolonged reaction time, which probably resulted from the TC diffusion into the internal adsorption sites of ferrihydrite. Thus, the overall adsorption rates of TC on ferrihydrite were slower than those on goethite.

### 3.5. Adsorption kinetics of tetracycline on Fe (hydr)oxides as affected by metal cations (Cu^2+^, Zn^2+^ and Al^3+^)

**Figure 4.**
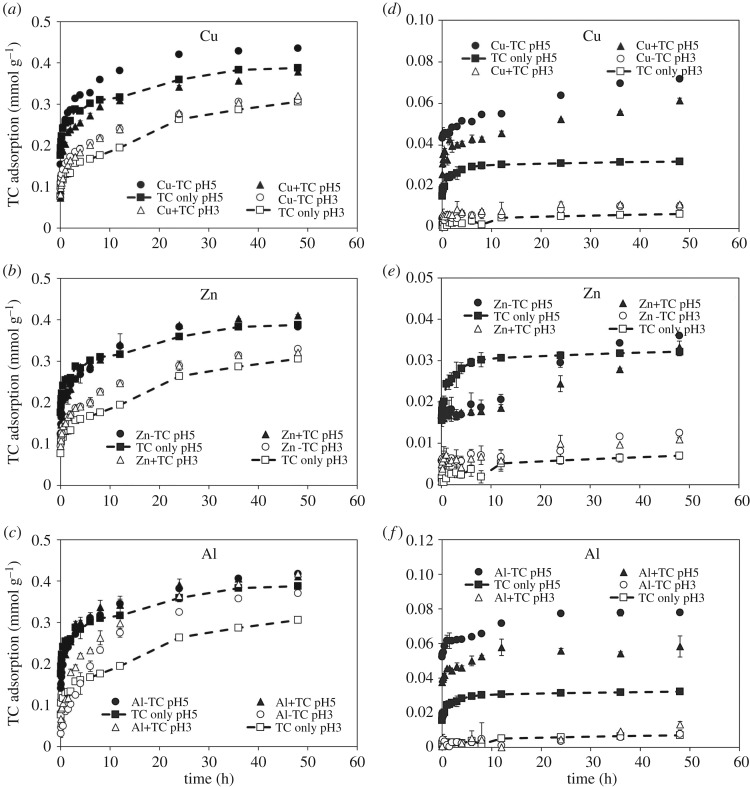
Adsorption kinetics of TC on (*a–c*) ferrihydrite and (*d–f*) goethite at pH 3 and 5 as affected by the addition of Cu, Zn and Al. The legends of metal–TC and metal + TC represent TC formed complexes with metals before the adsorption and metals reacted with absorbents prior to the TC addition. The data of TC only system were quoted from figure 3.

Metal cations of Cu^2+^, Zn^2+^ or Al^3+^ were added in the TC/Fe(hydr)oxide systems in two different ways: (i) TC and metals formed complexes before the adsorption on Fe (hydr)oxides (denoted as metal–TC in figure 4) and (ii) metals reacted with Fe (hydr)oxides for 1 h and then TC was added into the suspension (denoted as metal + TC in figure 4). For the adsorbent of ferrihydrite, while Zn^2+^ showed the least impact on the adsorption behaviour of TC compared with other metals (figure 4*b*), Cu^2+^ and Al^3+^ essentially promoted the TC adsorption at pH 5 and 3, respectively (figure 4*a*,*c*). For the Cu^2+^ that reacted with ferrihydrite prior to the addition of TC, the adsorbed Cu^2+^ showed negligible impacts on TC adsorption at pH 5 (figure 4*a*), suggesting that the formation of cationic bridge between TC and Cu^2+^ was impeded as a consequence of the lack of efficient charges or coordination sites on the adsorbed Cu^2+^. Unlike the adsorbed Cu^2+^, the adsorbed Al^3+^ on ferrihydrite still showed the complexation ability to TC at pH 3, increasing the TC adsorption on ferrihydrite (figure 4*c*). However, such enhancement in TC adsorption by Al^3+^ addition was less significant at pH 5. The possible explanation is the hydrolysis of Al^3+^ to Al(OH)^2+^/Al(OH)_2_^+^ might alter the structures or charges of Al–TC complexes.

On the adsorbent of goethite, the addition of metal cations generally showed less impacts for TC adsorption at pH 3 (figure 4*d*–*f*). Even if Al^3+^ could complex with TC at pH 3, a competition for the adsorptive sites on goethite between protons and Al–TC complexes may reduce the adsorbed amount of Al–TC complexes on the goethite surfaces (figure 4*f*). For the addition of Cu^2+^ and Al^3+^, both metal cations showed similar effects upon TC adsorption on goethite at pH 5 (figure 4*d*,*f*). The occurrence of Cu–TC and Al–TC complexes might result in the favourable electrostatic environments for the TC adsorption on goethite. By contrast to the ferrihydrite system, wherein the pre-added metal cations showed no increase in TC adsorption at pH 5, the pre-added Cu^2+^ and Al^3+^ on goethite still boosted the TC adsorption (figure 4*d*,*f*). Owing to the limited adsorption sites on goethite, the pre-added Cu^2+^ and Al^3+^ might present as both adsorbed and free cation species. Thus, such free Cu^2+^/Al^3+^ could complex with TC while TC was subsequently added, and form Cu–TC/Al–TC complexes. In terms of Zn^2+^, it exhibited an initial competition behaviour with TC for the surface sites on goethite (figure 4*e*); however, the affinity of TC on goethite may be greater than that of Zn^2+^, and thus TC adsorption increased accompanying a release of Zn^2+^ over the reaction time.

### 3.6. Adsorption of tetracycline on goethite as influenced by molar metal/TC ratios

**Figure 5.**
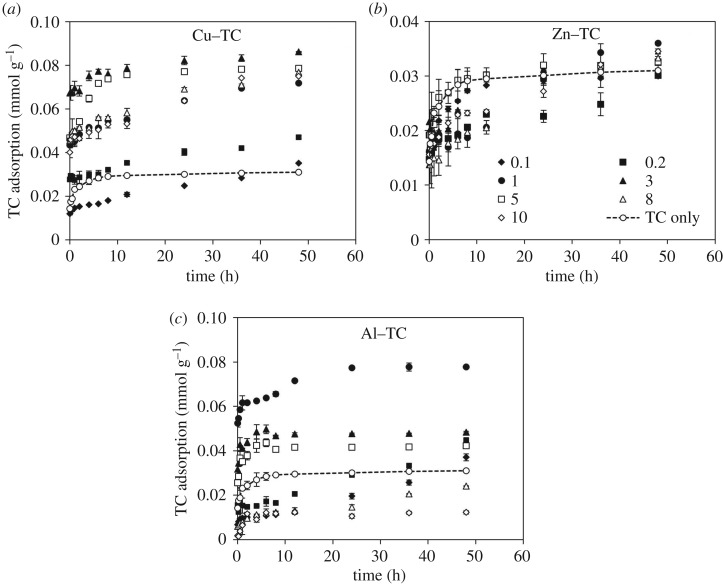
Adsorption kinetics of TC on goethite at pH 5 as affected by various addition of (*a*) Cu, (*b*) Zn and (*c*) Al. The adsorption experiments were conducted after TC formed complexes with metals. Molar metal/TC ratios ranged from 0.1 to 10 with fixed TC concentration of 0.0225 mM.

Although effects of metal cations on the TC adsorption have been widely studied [12,17,18,20,21,33], limited information was available regarding how the metal/TC molar ratio influences TC adsorption on Fe (hydr)oxides. Here, adsorption kinetics of TC on goethite at pH 5 as affected by metal/TC molar ratios ranging from 0.1 to 10 was investigated. Compared with the TC only systems (dashed lines in figure 5), TC adsorption was inhibited during the first 36 h of reaction in systems with Cu/TC molar ratio of 0.1, Zn/TC molar ratios of 0.1–10, or Al/TC molar ratios of 0.1, 8 and 10. The decreasing TC adsorption with Zn^2+^ addition (figure 5*b*) could be attributed to the competitive adsorption between free Zn^2+^ and TC as only negligible amounts of Zn^2+^ complexed with TC at pH 5. At the end of the reaction (48 h), the TC adsorption on goethite was eventually enhanced by the addition of Cu^2+^ and Zn^2+^, particularly the Cu^2+^ (figure 5*a*). Such results implied that the extra Cu^2+^/Zn^2+^ would not reduce the overall TC adsorption and suggested a greater affinity for Cu/Zn–TC complexes with goethite surfaces and/or the emergence of different sites on goethite for TC and Cu^2+^/Zn^2+^ adsorption. In the systems with Al/TC molar ratio ≥ 8, nonetheless, the TC adsorption was significantly suppressed (figure 5*c*). The possible interpretation was Al^3+^ or the hydrolysed Al(OH)^2+^ competed for adsorption sites of goethite with Al–TC complexes over the course of the entire reaction (figure 5*c*).

**Figure 6.**
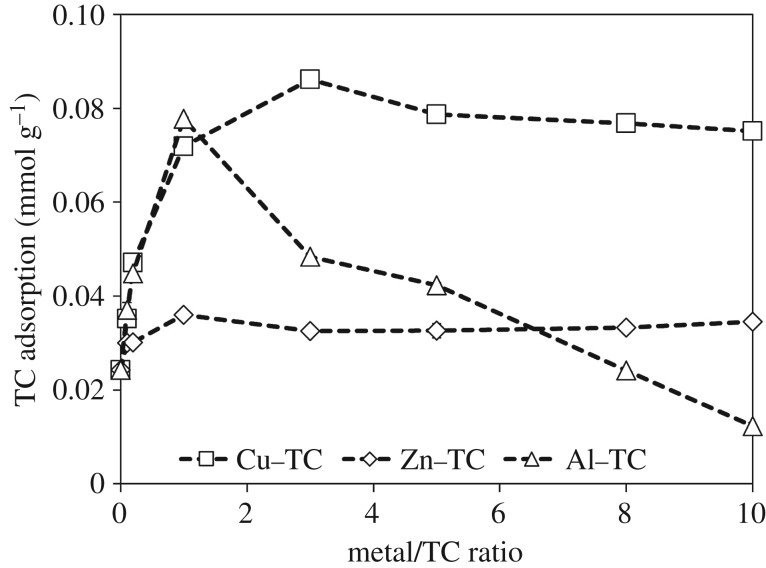
The relationship between adsorption capacity of TC after a 48 h equilibration at pH 5 on goethite and the added metal/TC molar ratios. The adsorption experiments were conducted after TC formed complexes with metals.

**Figure 7.**
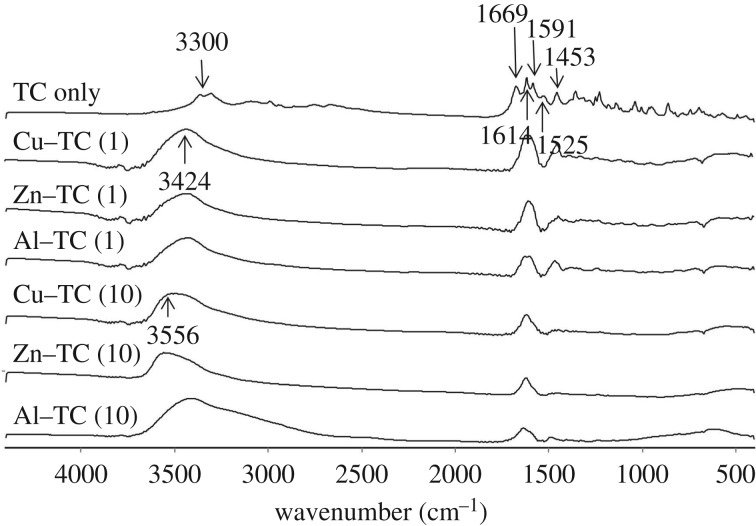
FTIR spectra for precipitate collected after a 48 h equilibration of TC adsorption on goethite at pH 5 as affected by the metal addition at molar metal/TC ratios of 1 and 10. The adsorption experiments were conducted after TC formed complexes with metals.

Amounts of adsorbed TC on goethite as influenced by the metal/TC molar ratios are shown in figure 6. Collectively, promoted TC adsorption by the addition of Cu^2+^ and Zn^2+^was found over the tested metal/TC molar ratios, although the addition of Zn^2+^ showed a less significant enhancement due to the relatively weak complexation with TC. The maximum TC adsorption in the Cu^2+^ and Al^3+^ system occurred at Cu/TC and Al/TC molar ratios of 3 and 1 (figure 6). A further increase in the Cu/TC and Al/TC molar ratio caused a slight decrease of TC adsorption in the Cu^2+^ system but a significant decrease in the Al^3+^ system. The formation of other Cu–TC species such as Cu_*n*_TC (*n* ≥ 2) or Cu(H)_*m*_TC_2_ (*m* ≤ 4) was less possible as it is difficult for one single TC molecule to bond two or more Cu cations [35] and such Cu/TC complexes were only formed under very acidic or alkaline condition [33]. Mitscher *et al*. [36] reported that Cu^2+^ tended to complex with the BCD ring of TC molecules (electronic supplementary material, figure S1) at pH < 7.5 with the Cu/TC molar ratio < 1. As the Cu/TC molar ratio increased to ≥ 1, however, the Cu^2+^ preferred to complex with the A ring of TC molecules, resulting in the shift for the maximum absorption of TC from 360–365 (Cu/TC < 1) to 378 nm (Cu/TC ≥ 1) [27]. Thus, an increase in TC adsorption with increasing Cu/TC molar ratio up to 3 may result partially from the changes in the complexation sites of TC for Cu^2+^ from BCD to A ring. We presumed that the conformations of the dominant species of Cu/TC complexes, i.e. CuHTC^+^, with a major bonding site of C1–C3 on A ring may closely match the steric arrangements of the adsorption sites on goethite [37]. Nonetheless, the preference for the adsorption of CuHTC^+^ on goethite was slightly inhibited as more Cu^2+^ was added, which might compete with the adsorption sites (figure 6). Al^3+^ may have a similar complexation behaviour with TC to Cu as evidenced by the red-shift of TC absorption in the UV–visible spectrum (electronic supplementary material, figure S2) and the peak shift for the amide group of TC in the FTIR spectrum from 3300 to 3424 cm^−1^ at a metal/TC ratio of 1 (figure 7). However, this amide peak remained unchanged as the Al/TC molar ratio further increased to 10, inconsistent with the Cu/TC and Zn/TC systems, wherein the amide group shifted to 3556 cm^−1^ at the molar ratio of 10. These results demonstrated that metal cations could complex with the C3 and amide groups on the A ring of TC even if the C11–C12 on the BCD ring of TC were the major bonding sites with a metal/TC molar ratio < 1 [23,33]. The preference of metal complexation with the BCD ring of TC was shifted to the A ring when the Cu/TC molar ratio increased, but it might not be the case in the Al^3+^ system with Al/TC molar ratio > 1. Thus, the excess Al^3+^ exhibited a substantial inhibition for TC adsorption due probably to the competitive effects between Al^3+^ and Al/TC complexes. In addition, we found that the excess Al^3+^ (Al/TC molar ratio > 1) could be specifically adsorbed on the goethite and increased the PZC of goethite from 6.5 to 7.5 (electronic supplementary material, figure S4). As a result, the positive charges on goethite surfaces increased, which may lead to an increase in the electrostatic repulsion between the goethite surfaces and Al/TC complexes.

## 4. Conclusion

TC and metals, such as Cu, Zn and Al, might distribute simultaneously in waters and soils via anthropogenic activities. The ubiquitous Fe (hydr)oxides in natural environments could serve as adsorbents and then control the dynamics and fate of TC. In this study, the maximum adsorption of TC on ferrihydrite and goethite occurred at pH 5–6, close to the pH_zpc_ of these two minerals, wherein a favourable electrostatic condition was created. Although the Zn^2+^ initially inhibited TC adsorption on Fe (hydr)oxides, the overall TC adsorption was not decreased due probably to the presence of different adsorption sites for Zn^2+^ and TC. Both Cu^2+^ and Al^3+^ could complex with TC and enhance TC adsorption through the formation of Fe (hydr)oxides–metal–TC ternary complexes. The presence of extra Al^3+^ could lead to a significant decrease in TC adsorptions; however, an excess of Cu^2+^ showed less of an impact on the TC adsorption on goethite. Spectroscopic results suggested that the complexation sites of TC with Cu^2+^ would change from BCD to A ring of TC molecules when the Cu/TC molar ratio increased to more than 1. The conformation of Cu/TC complexes with A ring of TC as the major binding site of Cu^2+^ may have a higher affinity to goethite surfaces, and thus the excess Cu^2+^ did not inhibit TC adsorption. However, the bonding sites for Al^3+^ on TC rings did not change, i.e. the structures of Al/TC complexes remained unchanged, along with various Al/TC molar ratios, and thus excess Al^3+^ would inhibit the adsorption of Al/TC complexes on goethite due to the competitive adsorption. Because the contents of polyvalent metal cations are generally higher than that of TC in natural environments, this study suggested that the molar ratios of polyvalent metals to TC and the ambient pH should be carefully considered while evaluating TC mobility in media enriched with Fe (hydr)oxides.

## Supplementary Material

Supplementary Material from “Adsorption of Tetracycline on Fe (Hydr)oxides: Effects of pH and Metal Cation (Cu2+, Zn2+, and Al3+) Addition in Various Molar Ratios”.

## Data accessibility

The additional experimental procedure, figures and tables supporting this article have been uploaded as part of the electronic supplementary material.
