# Supplementary material for: Adsorption of tetracycline on Fe (hydr)oxides: effects of pH and metal cation (Cu2+, Zn2+ and Al3+) addition in various molar ratios
Source: R Soc Open Sci. 2018 Mar 28;5(3):171941. doi: 10.1098/rsos.171941 (PMC5882719; doi:10.1098/rsos.171941)
Supplement: Supplementary Material from “Adsorption of Tetracycline on Fe (Hydr)oxides: Effects of pH and Metal Cation (Cu2+, Zn2+, and Al3+) Addition in Various Molar Ratios”. [file rsos171941supp1.pdf]

Supplementary Material

Adsorption of Tetracycline on Fe (Hydr)oxides: Effects of pH and Metal Cation (Cu^{2+}, Zn^{2+}, and Al^{3+}) Addition in Various Molar Ratios

Liang-Ching Hsu[a], Yu-Ting Liu[b*], Chien-Hui Syu[c], Mei-Hsia Huang[b], Yu-Min Tzou[b*], Heng Yi Teah[d]

[a] Scientific Research Division, National Synchrotron Radiation Research Center, 101 Hsin-Ann Road, Hsinchu 300, Taiwan

[b] Department of Soil and Environmental Sciences, National Chung-Hsing University, 145 Xingda Rd., Taichung 402, Taiwan

[c] Division of Agricultural Chemistry, Taiwan Agricultural Research Institute, No.189, Zhongzheng Rd., Wufeng Dist., Taichung City 41362, Taiwan

[d] Division of Environmental Studies, Graduate School of Frontier Sciences, The University of Tokyo, 332 Building of Environmental Studies, 5-1-5 Kashiwanoha, Kashiwa City, Chiba 277-8563, Japan

[*]Corresponding author:

Yu-Ting Liu: email: yliu@nchu.edu.tw; TEL: +886-4-2284-0373 ext. 3402; Fax: +886-4-2285-6050

Yu-Min Tzou: ymtzou@dragon.nchu.edu.tw; Tel.: +886-4-2284-0373 ext. 4206; fax: +886-4-228-5516

Preparation and characterizations of Fe (hydr)oxides

The suspensions of goethite and ferrihydrite were dialyzed until the electric conductivity was < 50 μS/cm^{-1}. Final stock suspensions in 0.01M NaCl background were stored at 4 ^{o}C for a maximum of two weeks.

The X-ray powder diffraction analysis was conducted using the PANalytical X'Pert Pro MRD instrument with a step of 0.017° in a 2Ɵ range from 15° to 90° with the Cu-Kα radiation (λ = 1.5406 nm, 40 kV, 40 mA). The point of zero charge was determined using the pH drift method [1]. The average particle sizes were measured by dynamic light scattering (Malvern/Nano-ZS, USA), and the specific surface areas were determined by N_2-BET method.

TC Adsorption on Fe (hydr)oxides

To avoid the possible photolysis of TC, the reaction vials were covered with Al foil and placed in a stainless steel chamber during the equilibration. Control experiments conducted at each tested pH in the absence of adsorbents showed that < 1% of TC was lost, implying the negligible photolysis of TC over the course of incubation. All experiments were performed in three replicates.

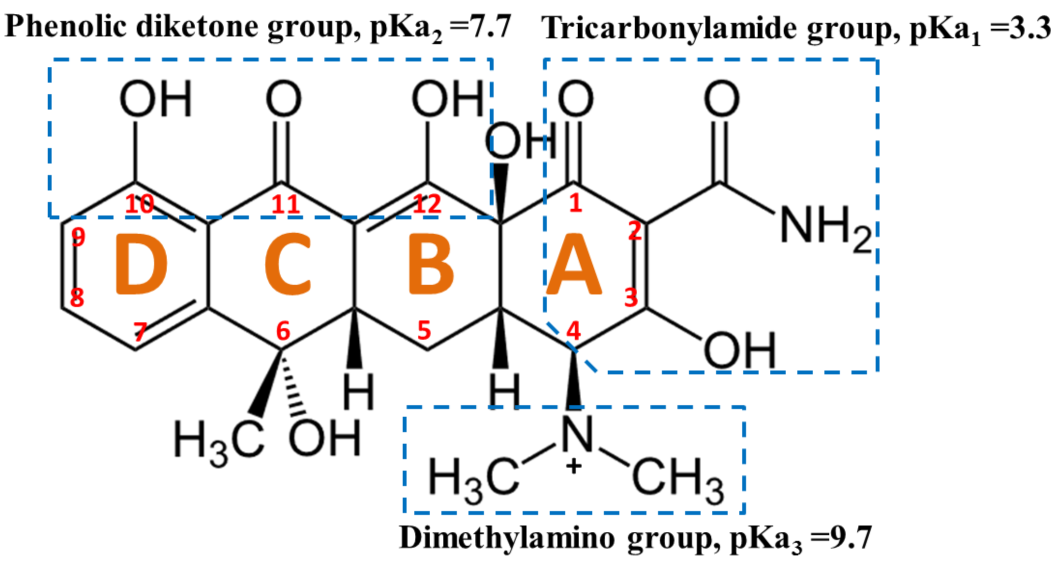

Figure S1. The molecular structure of tetracycline (TC) and dissociation constants (pKa) of the dominant acidic groups.

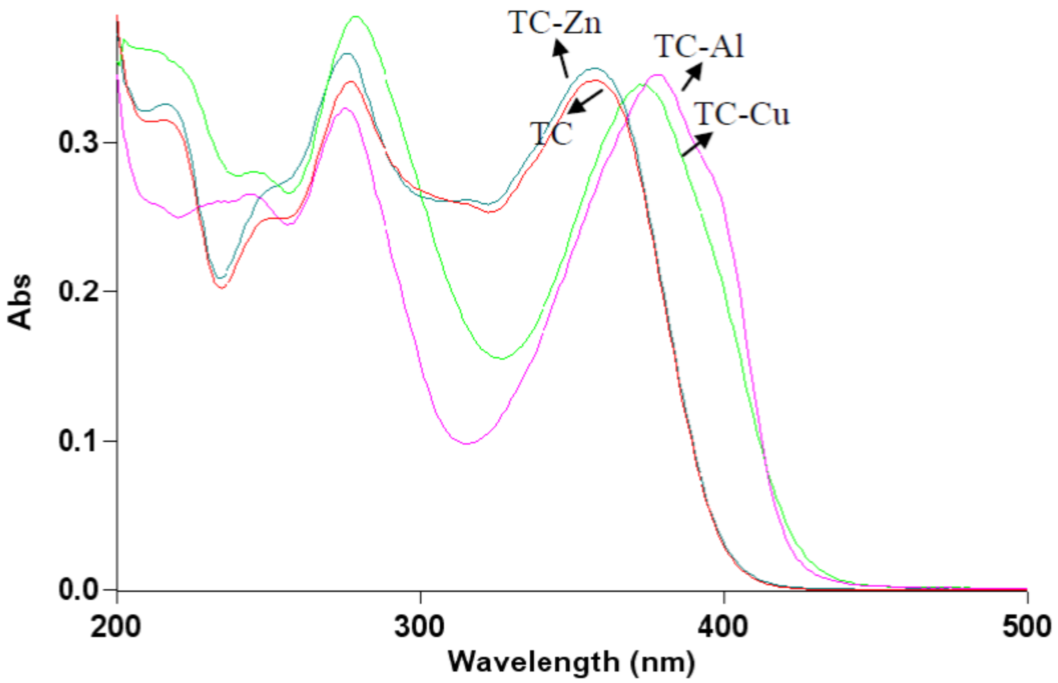

Figure S2. The UV/Vis absorption spectroscopy of TC and TC-metal complexes formed at the metal to TC molar ratio of 1 : 1 (each 0.0225 mM) for 24 h.

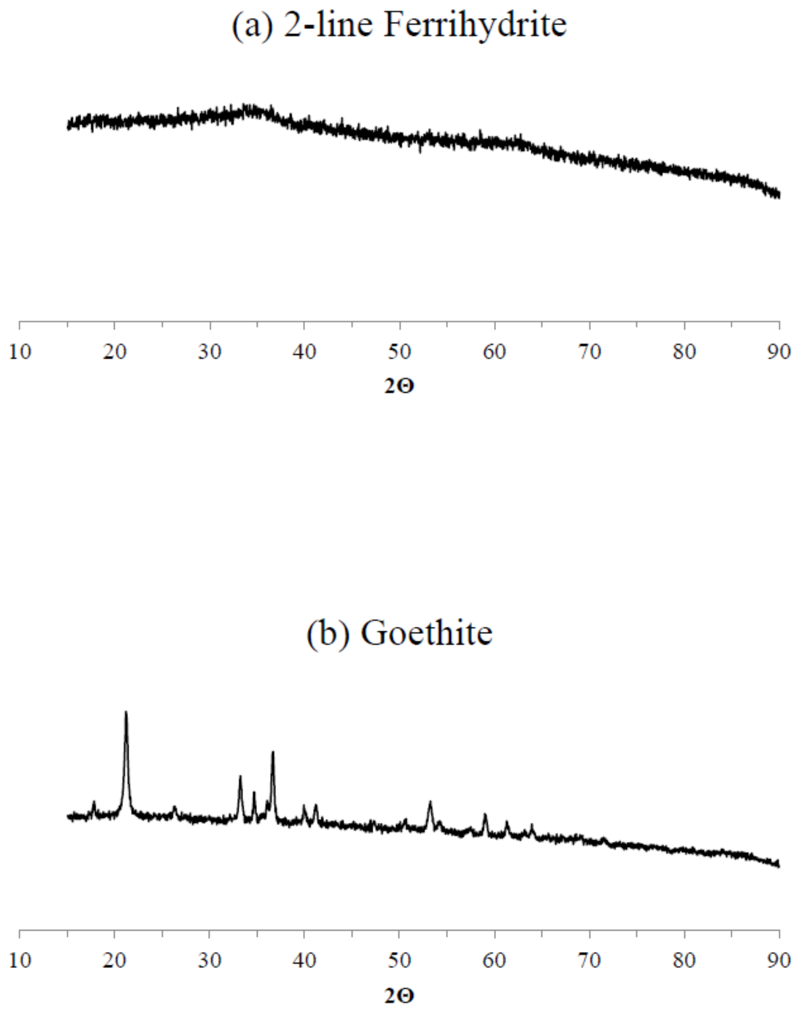

Figure S3. X-ray diffraction patterns of (a) 2-line ferrihydrite and (b) goethite

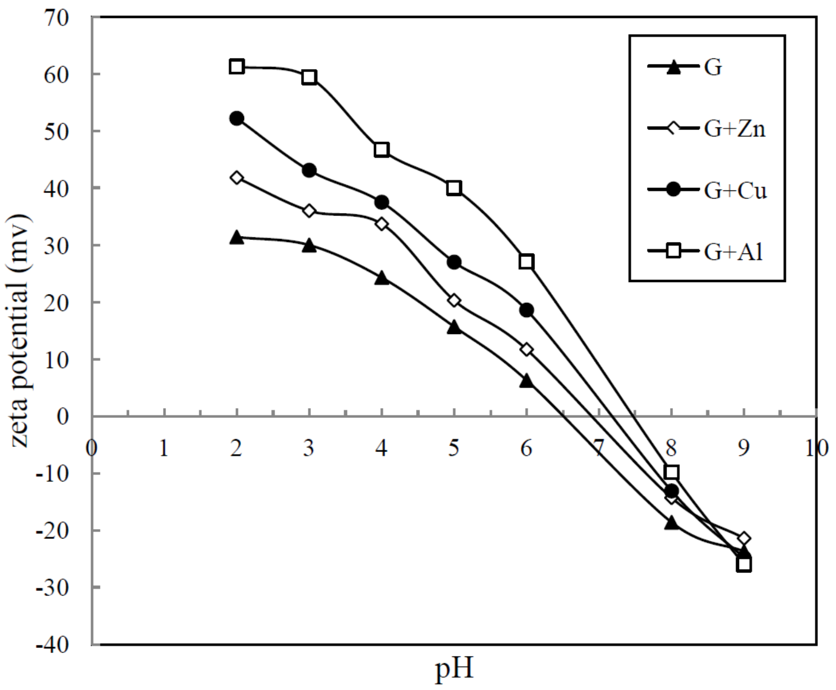

Figure S4. Effect of metals on the zeta potential of goethite.

Table S1. Characteristics of the Fe (hydr)oxides used in this study.

Iron hydroxide	pH_{PZC}	Particle size nm	Surface area $m^2\ g^{-1}$
2-line Ferrihydrite	6.3	290.3	251.8
Geothite	6.5	466.5	25.0

Table S2. Parameters of Freundlich isotherm model for TC adsorption on ferrihydrite and goethite.

		Ferrihydrite			Goethite		
		K_f	1/n	R^2	K_f	1/n	R^2
TC only	pH 3	1.202	0.222	0.991	0.141	0.400	0.980
	pH 4	2.079	0.318	0.962	0.300	0.833	0.993
	pH 5	2.140	0.232	0.990	0.387	0.700	0.940
	pH 6	2.763	0.288	0.983	0.759	0.855	0.991
TC+Cu^{2+}	pH 3	0.947	0.194	0.955	0.958	0.945	0.964
	pH 4	1.531	0.248	0.973	1.013	0.857	0.988
	pH 5	2.866	0.346	0.996	1.514	0.709	0.986
	pH 6	8.116	0.418	0.993	4.113	0.766	0.987
TC+Zn^{2+}	pH 3	1.111	0.227	0.973	0.208	0.688	0.868
	pH 4	1.876	0.313	0.975	0.328	0.787	0.968
	pH 5	2.261	0.306	0.977	0.457	0.661	0.972
	pH 6	2.839	0.282	0.957	0.999	0.820	0.986
TC+Al^{3+}	pH 3	1.288	0.199	0.996	2.241	1.167	0.958
	pH 4	2.198	0.257	0.999	1.665	1.003	0.963
	pH 5	2.400	0.265	0.957	0.751	0.704	0.956
	pH 6	1.886	0.203	0.980	1.119	0.716	0.961

Freundlich isotherm equation: $q_e = K_f Ce^{1/n}$; where qe is the amounts of TC adsorption onto Fe (hydr)oxides (mg g^{-1}); Ce = equilibrium concentrations of TC (mmol L^{-1}); K_f = Freundlich isotherm constants ($mmol^{1-1/n}$ $L^{1/n}$ g^{-1}); n = adsorption intensity .

Table S3. Pseudo-second order parameters for TC adsorption on ferrihydrite and goethite at pH 3, 5, and 8.

	Ferrihydrite			Goethite		
	k	q_e	R^2	k	q_e	R^2
	g $mmol^{-1}$ h^{-1}	mmol g^{-1}		g $mmol^{-1}$ h^{-1}	mmol g^{-1}	
pH 3	0.691	0.298	0.902	29.25	0.007	0.900
pH 5	2.351	0.392	0.906	47.33	0.032	0.999
pH 8	1.340	0.287	0.940	3.69	0.015	0.841

Pseudo-second-order equation: $dq_t/d_t = k(q_e-q_t)^2$, where k is the rate constant of adsorption; q_e is the amounts of TC adsorption onto Fe hydr(o)oxides at equilibrium; q_t is the amounts of TC adsorption onto Fe hydr(o)oxides at any time.

Reference:

[1] C. Sun, J.C. Berg, A review of the different techniques for solid surface acid–base characterization, Adv. Colloid Interface Sci. 105 (2003) 151-175.
